# Implementing Universal and Targeted Policies for Health Equity: Lessons From Australia

**DOI:** 10.34172/ijhpm.2021.157

**Published:** 2021-11-09

**Authors:** Matthew Fisher, Patrick Harris, Toby Freeman, Tamara Mackean, Emma George, Sharon Friel, Fran Baum

**Affiliations:** ^1^Southgate Institute for Health, Society and Equity, Flinders University, Adelaide, SA, Australia.; ^2^Centre for Primary Health Care and Equity, University of New South Wales, Sydney, NSW, Australia.; ^3^College of Medicine and Public Health, Flinders University, Adelaide, SA, Australia.; ^4^The George Institute for Global Health, Sydney, NSW, Australia.; ^5^School of Allied Health Science and Practice, University of Adelaide, Adelaide, SA, Australia.; ^6^RegNet School of Regulation and Global Governance, Australian National University, Canberra, ACT, Australia.

**Keywords:** Universal Policy, Targeted Policy, Equity of Access, Social Determinants, Indigenous Health, Australia

## Abstract

**Background:** Debate continues in public health on the roles of universal or targeted policies in providing equity of access to health-related goods or services, and thereby contributing to health equity. Research examining policy implementation can provide fresh insights on these issues.

**Methods:** We synthesised findings across case studies of policy implementation in four policy areas of primary healthcare (PHC), telecommunications, Indigenous health and land use policy, which incorporated a variety of universal and targeted policy structures. We analysed findings according to three criteria of equity in access – availability, affordability and acceptability – and definitions of universal, proportionate-universal, targeted and residual policies, and devolved governance structures.

**Results:** Our analysis showed that existing universal, proportionate-universal and targeted policies in an Australian context displayed strengths and weaknesses in addressing availability, affordability and acceptability dimensions of equity in access.

**Conclusion:** While residualist policies are unfavourable to equity of access, other forms of targeting as well as universal and proportionate-universal structure have the potential to be combined in context-specific ways favourable to equity of access to health-related goods and services. To optimise benefits, policies should address equity of access in the three dimensions of availability, affordability and acceptability. Devolved governance structures have the potential to augment equity benefits of either universal or targeted policies.

## Background

**Key Messages**

** Implications for policy makers**

- Theory-informed analysis of policy implementation can show governments how to combine universal and targeted policies to improve equity of access to healthcare and other health-related goods and services.
- With the exception of ‘residualist’ approaches to targeting, both universal and targeted strategies have the potential to support equity in availability, affordability and acceptability of health-related goods and services.
- Combinations of universal and targeted approaches will be best placed to support equity when implemented in ways that respond to context.
- Policy-makers should consider use of devolved governance structures operating regionally, to augment equity benefits of either universal or targeted policies.

** Implications for the public**

Governments’ policies do much to determine people’s access to services and infrastructure relevant to health such as healthcare, telecommunications or a healthy built environment. Equitable access to such goods and services, according to need, will support equity in health outcomes. Universal policies aim to ensure equal access for all citizens to a particular good or service; targeted polices aim to meet the needs of a specific population group. In this article we examine how governments can combine universal and targeted policy approaches, and use devolved forms of policy governance, to improve equity of access to services and infrastructure relevant to health; including for Indigenous peoples.

Policy-makers and researchers have long debated the merits of universal or targeted policy strategies in relation to the role of the welfare state and citizen’s rights,^1,2^ or to achieve goals such as poverty reduction,^3^ universal health coverage^4^ or health equity.^5^ The World Health Organization (WHO) Commission on Social Determinants of Health recommended a mix of universal and targeted policies.^6^ However, significant questions remain about how universal or targeted policies, or a combination of these, can be deployed to improve health equity effectively in particular contexts.^7^ These questions remain important in a period in which health inequities have grown in Australia^8^ and other countries,^9,10^ and life expectancy has fallen for some groups subject to socioeconomic disadvantage.^11^ Here we draw on research on policy implementation in four areas of Australian policy – Indigenous health, primary healthcare (PHC), telecommunications and land use planning (LUP) – to assess the role of universal and targeted policies, and devolved governance structures, in supporting health equity.

 As a national political principle, universalism asserts that citizens are entitled to goods and services to meet basic needs such as healthcare, education or housing.^1^ After World War Two, the UK Beveridge Report^12^ led to universalist social policies in areas such as healthcare and unemployment protection; replacing targeted welfare programs for those deemed to be most disadvantaged.^13^ Since the 1980s, the rise of neoliberal politics favouring reduced state intervention in capitalist markets^14^ has seen some retreat from universalism and revival of selective, targeted approaches.^3^

 In public health, governments’ use of universal or targeted policies are understood to affect health equity by determining equity of access to social determinants of health (SDH) such as healthcare, housing, education, and infrastructure.^5,6,15^ Furthermore, all policy sectors are seen to have a role in healthy public policy.^16^ Although universal social policies are commonly seen as favourable to population health and health equity,^17,18^ their ‘one-size-fits-all’ approach may fail to meet the specific needs of particular population groups,^19^ especially if underlying differences in access to SDH are in place, and targeted policies may be a more effective way to meet these needs and reduce associated health inequities.^20,21^

 Furthermore, public health literature has extended on categories of ‘universal’ and ‘targeted’ to introduce concepts such as ‘proportionate universalism,’^22^ which call for a combination of universalism and targeting, to deliver universal services but ‘at a scale and intensity proportionate to the degree of need.’^23^ Others have pointed out that merits of targeted social policies as a means to address health inequities may depend on whether they are combined with universal policies or, alternatively, used in combination with market structures where, apart from the targeted group, access to the relevant good or service depends on individuals’ private ability to pay.^13^

 Further research is needed to examine empirically and in more depth the advantages and disadvantages of various universal or targeted policies, or combinations of these, to achieve a goal of healthy public policy in practice,^24^ and contribute to gains in health equity.^13^ Within a larger project examining Australian policy responses to SDH equity,^7^ we conducted four case studies of policy implementation – in areas of telecommunications, PHC, Indigenous affairs and LUP – encompassing various universal and targeted structures. Our case studies examined structures and processes of policy implementation to identify how they affected equity of access to health-related goods or services provided via universal or targeted services, and considered implications for population health.^25-28^ In Australia, health inequities are shaped by socioeconomic status (SES) and differences in access to services between major urban centres and other regional, rural or remote locations.^29^ Indigenous peoples in Australian are subject to significant health inequities, caused by inequitable access to SDH, exposure to racist policies and loss of self-determination; all of which have their roots in on-going processes of colonisation.^30^

 Proceeding from the above, this paper addresses the following research question: What lessons emerge from a synthesis of findings from our four case studies for how governments can implement universal and targeted policy structures, so as to improve equity of access to health-related goods and services? Responding to our findings, we focus on devolved governance structures as a potential means to implement universal or targeted policies,^24^ bringing a fresh perspective to the key question of how to implement universal policies in ways that also meet the needs of particular groups. While our focus is on Australia, results hold salient lessons for policymakers in other, similar jurisdictions.

## Methods

 We selected four areas of contemporary Australian policy for study based on the SDH themes of macroeconomics and infrastructure, health systems, Indigenous affairs, and land use and urban environments. These policies were, respectively, National Broadband Network (NBN) policy, PHC policy, ‘Closing the Gap’ (CTG) Indigenous health policy, and LUP policy in Sydney, Australia’s largest city. The first three of these were national policies; the fourth occurred under the aegis of one (sub-national) State Government, working in collaboration with the national government.

 We conducted a qualitative case study of implementation in each policy area; a methodology well-suited to examining complex phenomena in real world settings.^31^ In each of these four case studies we applied methods of mapping policy structures, monitoring grey literature to track policy debate and change, and semi-structured key informant interviews held with senior level policy actors from relevant government agencies or non-governmental organisations, or independent experts. In each case we used purposive and snowball sampling to identify and recruit interviewees. Details on these methods have been published elsewhere.^25-27,32^ We conducted 86 interviews across the four cases.

 To inform the data collection and analysis for individual case studies we drew on theory related to SDH^33^ and political science theory on the role of ideas, actor-interests and institutional structures in shaping policy,^34^ as well as theory on multi-level governance^35,36^ and policy implementation.^37^ Thematic analysis of interview data was conducted with a coding framework based on these theoretical concepts, using NVivo software. Theory on multi-level governance argues that devolving some authority from central public agencies to governance structures operating at a regional or local scale may have benefits for policy delivery by enabling flexibility to adapt services to suit local conditions and needs.^36^ This thinking has parallels in public health literature identifying potential benefits of ‘place-based’ policy and healthy cities strategies, to respond flexibly to population health needs within a particular locale.^38-40^

 Implementation theory highlights the intersecting roles of public agencies and governance structures, and private sector or not-for-profit organisations in determining policy outcomes.^37^ In the CTG policy – focused on addressing health inequities affecting Aboriginal and Torres Strait Islander peoples – we also drew on evidence on social determinants of Indigenous health,^30^ and applied a framework to assess cultural safety in policy.^41^

 We analysed data to assess key factors shaping policy implementation, in ways likely to affect equity of access to healthcare and other SDH. We did not seek to assess policy outcomes. The interview data were analysed with the assistance of NVivo software. All case studies received ethics approval from the Flinders University Social and Behavioural Research Ethics Committee.

 Having completed these four case studies, through a process of inductive analysis and comparison of findings across cases, we concluded provisionally that our findings, considered as a whole, offered evidence relevant to assessing the role of universal and targeted policies in contributing to healthy public policy and health equity. The data in the cases suggested that universal and targeted structures were the mechanisms by which equity was impacted. We therefore chose to use those theories to critically explain those structural dynamics in the dataset within and across the cases. This is a mix of inductive (data from the cases) and abductive (applying critical theoretical perspectives on policy to further test and explain that inductive data) reasoning following critical social science methodology.^42^ To develop this paper, the authors then used team discussions to triangulate data from across our four case studies on the structural features of policy implementation, and analyse these inductively according to: (*a*) theoretical perspectives on equity of access to healthcare, other social services, telecommunications infrastructure and healthy built environments; and (*b*) definitions of universal, proportionate-universal, targeted and residual policies, and devolved governance structures. The fact that the four areas of policy examined are not overtly about the same policy problems, in our view, presents no disadvantage to our approach. On the contrary, they provide evidence concerning application of universal and targeted approaches in public policy, and of devolved governance structures, across several different policy sectors; each of which affects equity of access to SDH.

 Drawing on literature on access to healthcare^43^ and on health equity,^44^ we defined equity of access as a situation where all people are easily able to use health-related goods and services in ways that meet their needs, regardless of underlying inequalities in economic or social resources. Although there are other frameworks defining up to five dimensions of equity of access to healthcare,^45^ we took the view that Thiede and colleagues’ three-part framework^46^ is more parsimonious and better suited to our broader considerations of equity of access to health-related goods and services. We recognised that equity of access so defined is affected by three main aspects of how goods or services are provided^46^:

- **Availability**: concerned with goods or services being available in the place and time they are needed; including regional, rural or remote areas;
- **Affordability**: concerned with the degree of fit between the costs of goods or services and individual’s ability to pay, and;
- **Acceptability**: concerned with the capacity of goods or services to meet a variety of needs related to factors such as age, gender, ethnicity, Indigeneity, language, cultural beliefs, location, income, employment status or education.

To conduct our analysis of structural policy features we applied the following definitions:

- **Universal** policies aim to ensure all residents or all citizens (or all members of a broad population group; eg, women, men, children, older people) have equal access to a baseline level of a good or service, commonly taking into account considerations of availability and affordability.^13^
- **Proportionate-universal** policies aim for universal access to a service but resource and deliver those services at a scale and intensity proportionate to assessed differences in population needs across levels of SES, within differing population groups, or in differing locations.^22,23^
- **Targeted** policies aim to provide access to a good or service intended to meet particular needs or goals of a specified population group. Groups may be defined according to criteria such as SES, health-related needs, indigeneity, ethnicity, gender, sexuality, migrant or refugee status, disability or geographic location; or a combination of these. Targeted strategies may be implemented on their own, or combined with universal policies.^13,47^
- **Residualist**policies are a form of targeting, where public funding is used to provide (or subsidise) access to a good or service for specified groups deemed to be particularly disadvantaged or otherwise in need, in an area of policy where otherwise access to that good or service depends wholly on private individual/family ability to pay.^13^ While targeting can be and is used in combination with universal public policies, residualist policies, by definition, are instead of universal policies.
- **Devolved governance structures **in Australia are national or State government policies that assign (some) authority to a governance structure operating within a particular sub-region of a State jurisdiction, to manage the way policy resources are deployed, in a way that is tailored to population needs within that area.^36^

Sections below also include discussion of comprehensive primary healthcare (CPHC), which we define as first-level care that incorporates but extends beyond primary medical care to include multi-disciplinary care, health promotion, disease prevention, community engagement and action to address SDH.^25^

## Results

 The four areas of policy examined differed in the extent to which they addressed the three dimension of equity of access: availability, affordability and acceptability. The performance of each sector is summarised in Table and is described in more detail in the analyses to follow where, after a summary of case studies, we set out our findings; firstly in relation to universal policies and then considering proportionate-universal policies, targeted policies and finally devolved governance structures.

**Table:** Policy Areas’ Performance on Three Dimensions of Equity in Access

	**PHC**	**NBN**	**CTG**	**LUP**
Availability	✕	✓	✓	✓
Affordability	✓	✕	✓	✕
Acceptability	✕	✕	✓/✕	✕

Abbreviations: PHC, primary healthcare; LUP, land use planning; NBN, National Broadband Network; CTG, Closing the Gap. *Key:* ✓= good performance, ✕ = weak performance, ✓/✕ = mixed.

###  Case Studies

 The four areas of policy examined in our research are outlined in Box 1, highlighting key structural features identified in our research as relevant to equity outcomes.

**Box 1. Case Study Policy Areas and Structural Features Relevant to Equity of Access**

** Primary Healthcare**

- Medicare: a universal, national public insurance scheme. Subsidises access to primary medical care, mainly delivered by private GPs.
- Medicare subsidies may cover the whole of service users’ costs for visiting a GP, but GPs can also charge additional fees.
- Medicare is not available to some groups without citizenship or permanent residency.
- Medicare-funded providers are not subject to constraints on where to practice. There is a concentration of services in high-income, low-need, inner urban regions.^29^
- Federal or State governments fund additional targeted PHC services: eg, child and maternal health, alcohol and drug, Indigenous health, mental health, public dental.
- Federal government funds regional PHNs for population health planning, service coordination and commissioning to fill service gaps. PHN funding is proportionate based on indicators of need by PHN region (either sub-regions, or the whole area of State government jurisdictions).
- PHI system covering non-Medicare PHC services: eg, dental, physiotherapy and psychology. Approximately 50% of adults hold some form of PHI; Federal government provides $11 billion in direct and indirect public subsidies per year.^48^

** ‘Closing the Gap’ **

- CTG policy aims to reduce or eliminate ‘gaps’ in health, education and employment outcomes between Aboriginal and Torres Strait Islander peoples and non-Indigenous Australians.
- Includes targeted funding for stand-alone services and targeted strategies within universal healthcare, education and employment services.
- A mix of Medicare and targeted funding supports 140+ ACCHOs delivering comprehensive PHC.
- Targeted funding for public Aboriginal Medical Services in rural/remote areas.
- Many other targeted health or social service programs.
- Some Aboriginal community-led governance structures partner with government agencies to implement policies.

** Land Use Planning**

- New South Wales State government created the GSC, to coordinate urban planning and infrastructure investment in Sydney.
- GSC acts as lead agency in the WSCD agreement, involving Federal and State and Local government agencies.
- Western Sydney a socially and economically disadvantaged region; home to around 2.5m people. Problems of urban sprawl, poor access to services and public transport, limed employment opportunities.
- WSCD aims to leverage infrastructure investment to create a ‘nodal’ Western Sydney city and improve access to employment, services and infrastructure.

** National Broadband Network**

- Digital telecommunications mediate access to determinants of health such as employment, education and social contact.^26^
- NBN is a national policy for universal access to basic HSB via new or re-purposed telecommunications infrastructure.
- NBN Co is a government owned private company; installs and owns NBN infrastructure; sells wholesale access to private companies operating in a competitive, retail market.
- Private companies on-sell fixed-line phone and internet services to citizens; graded costs structure based on data speeds.
- Universal wholesale pricing mechanism intended to equalise costs across regions.
- Different NBN delivery technologies offer different performance capabilities.
- Higher income areas more likely to have best performing technologies^49^; potential redundancy of weaker performing or older technologies as data demands increase.
- Little effort from Department of Communications to engage other sectors (eg, health) to ensure infrastructure meets needs.

------------------

Abbreviations: PHC, primary healthcare; LUP, land use planning; GP, general practitioner; PHN, Primary Health Network; ACCHOs, Aboriginal Community Controlled Health Organisations; GSC, Greater Sydney Commission; WSCD, Western Sydney City Deal; NBN, national broadband network; HSB, high speed broadband; PHI, private health insurance

###  Universal Policies

 Two policy areas – PHC and NBN – were committed to universal access.^25,26^ In both cases, policies were implemented in a manner consistent with our definition, to provide universal access to a baseline level of services. In both cases, the nature of the defined baseline and specific structures used to implement the policies had mixed effects on equity of access, as discussed below.

 In PHC policy, Medicare is the central feature, subsidising access to primary medical care, delivered mainly by GPs.As an informant described, ‘*compared to many other countries Australia does very well … *w*e have … universal health coverage through Medicare [offering] subsidised, and in many cases free, access to GP services*.’ Medicare supports equity of access to primary medical care across levels of SES^50,51^ and this compares favourably with a residualist system in primary dental care; where access depends on tightly targeted, resource-poor public services, or on private health insurance (PHI) and out-of-pocket payments. This leads to significant inequities in timely access to dental care and in health outcomes between those with or without PHI.^52,53^ As one informant noted, ‘*if you want an example of a two tiered system we’ve got it in dental.’*

 However, the structures of Medicare – focused on affordable access to episodic primary medical care as the ubiquitous baseline model – failed to address other differences salient to health equity. We identified three main issues. First, the baseline model of episodic primary medical care is a poor fit for people with chronic or non-communicable diseases^54^ (more common among lower income groups and Aboriginal and Torres Strait Islander people), and fails to meet the specific health or service needs of other groups. As an informant noted, ‘*the needs of people … where there are real inequities in access or outcomes are not the ones that need just an episodic-type management.’ *Our case study findings indicated that a CPHC model as the baseline service would be better placed to meet the needs of people with non-communicable diseases and other groups.^25^

 Second, universal PHC services were recognised as creating barriers to access for Aboriginal and Torres Strait Islander service users because of racist practices and attitudes. As one participant commented, ‘*certainly there are [mainstream] service providers that … do build cultural competency and safety into their service provision … [but] the big issue we have is racism; that is front and centre*.*’* This is a clear failure in acceptability.

 Third, because the funding structures for general practitioner (GP) services do not regulate location, there is a concentration of services in inner-urban areas of major cities where income is higher and need is less, and inadequate availability in the outer suburbs, regional towns and rural and remote areas with lower incomes and greater needs.^29^ Furthermore, spending on PHC services per person per year is highest in inner urban areas and progressively reduces in outer suburban, regional, rural and remote areas.^55^ As an informant described, *‘in rural and … remote areas there is an undersupply of the workforce … and an underutilisation of Medicare*.*’*

 So, while Medicare as a universal policy delivers reasonably well on affordability, the limited baseline model of service and inequitable geographic distribution of services creates problems for equity of access in dimensions of acceptability and availability.

 NBN policy was originally planned to replace ageing telecommunications infrastructure and deliver universal access to affordable, high-quality high-speed broadband services. As a key policy actor involved at the time said, ‘*…telecommunications should be considered a utility in the same way as … power [or] water … The fundamental issue around a utility is the quality of access*.*’ *Again, universality was seen as better for equity of access than a purely market-based or residualist approach. As one informant put it, ‘*left to the market, areas of disadvantage, less accessible areas, will be left to last or will be under-serviced.’ *

 Initially, NBN implementation used high-performing fibre-optic cable as the main delivery technology. However, a new government in 2013, while retaining a commitment to universality, set a low standard for baseline service quality and switched to a mix of technologies with different performance capabilities.^26^ Subsequently, higher-income areas tended to get better-performing technologies while lower income areas got those with lesser performance.^49^ Furthermore, the mixed public-private structure of NBN implementation has resulted in poor affordability, compared with other OECD (Organisation for Economic Co-operation and Development) countries. As reported previously, we found agencies conducting NBN implementation did little to engage with other policy sectors, local communities or groups with specific needs to ensure the infrastructure met a variety of needs.^26^

 Thus, while NBN policy did well on universal availability of a baseline quality of service (which is better for equity than a pure market or a residual model), it did less well on equity in affordability and acceptability.

###  Proportionate-Universal Policies

 In our case studies only one instance of proportionate funding was evident: funding to regional PHC bodies – primary health networks (PHNs) – is weighted according to a combination of measures of population size, rurality and socioeconomic factors. The latter two can be regarded as proxy indicators of healthcare need. While at the time of our research PHNs were quite new and their funding limited, they were seen to have potential to implement universal PHC in a way proportionate to need in different regions. As one informant said, ‘*I think that regionalisation is a fantastic way of building at least geographic equity into our system … their funding is weighted for rural and remoteness, which is a great start*.*’*

 Although there are significant constraints on the way PHNs operate currently (discussed below), proportionality in funding could address some of the geographic inequities in Medicare noted above.

###  Targeted Policies

 We found targeted policies were implemented in three main ways:

1. To fund residual public services (eg, as per dental care, described above).
2. To provide stand-alone services meeting the needs of a particular group, operating *alongside* universal services within the same broad area of policy (eg, specialised alcohol and drug services operating within the PHC sector).
3. As separately funded targeted programs, implemented *within* the structures of universal policies (eg, a quit-smoking program, implemented by GP services).

All three kinds of targeting were evident in our PHC and CTG cases including specialised, targeted services in areas such as dental health, child and maternal health, sexual health, family health, mental health, alcohol and other drugs, Aboriginal and Torres Strait Islander health and health promotion. We judge that the latter two approaches constitute two distinct forms of universal-plus-targeted policy.^47^

 As an example of the first universal-plus-targeted approach (point 2 above), long-standing concerns about availability and acceptability of universal services led Aboriginal and Torres Strait Islander communities to establish 140+ stand-alone Aboriginal community-controlled health organisations (ACCHOs), which provide culturally safe services. ACCHOs and a relatively small number of other community health services combine Medicare funding (for primary medical care) with multiple sources of targeted funding, to deliver a CPHC model of service. These services operate alongside the ‘mainstream,’ universal PHC system.

 In our PHC case, apart from residual policies, the combination of universal primary medical care with an array of targeted policies could be seen as addressing some of the weaknesses in availability and acceptability found in the universal system. Other examples include Federal target funding to encourage GPs to operate in under-served rural areas (availability), and States funding for migrant and refugee PHC services (acceptability and possibly availability).

 In our CTG case study a suite of targeted policies in the health, education and employment sectors were intended to reduce or eliminate inequities in health, education and employment outcomes between Aboriginal and Torres Strait Islander people and other Australians. All could be seen as fitting one or other of the two forms of universal-plus-targeted policy above. For example, the Federal Health agency funds ACCHOs to operate alongside generalist primary medical care services, and also funds programs to improve the cultural safety of universal services. Again, both strategies might be seen as augmenting the equity performance of the health sector overall.

 However, within both the PHC and CTG case studies we also identified a range of problems in how targeted polices were implemented, all relating to the crucial implementation relationship between government regulatory agencies and funded service providers.^37^ Triangulation of findings across these two case studies found a fragmented array of many small targeted policies in two levels of government, which often had one or more of the following characteristics:

- Programs focused on narrowly defined strategies, limited to specified actions to address specified problems (eg, treating patients at high risk of heart disease).
- Short-term, insecure funding, influenced by frequent (politically motivated) policy change.
- A top-down, prescriptive ‘performance management’ approach^37^ to regulation and reporting.

As one participant (with much policy experience) described:

*“[Governments have] maintained this funding approach where it’s ‘we’ll fund that program, then we’ll stop that program. We’ll fund this program’ and I suggest that a lot of that is based on the wanting to announce other policy.” *

 One of the resulting challenges for many (but not all) specialised PHC services was having to continually dedicate significant resources to seeking further funding, detracting from services offered.

*“20 [to] 40 percent of the resources of some of these small organisations that are doing critical work are redirected to grant processes directed by government. Imagine if that money was spent on actually helping the people that they’re there to help.” *

 Another challenge identified was a poor fit between prescriptive, one-size-fits all targeted programs and actual needs within a particular community. As one informant from our CTG case study noted:

*“…you might get funding for ear health, right, whereas in [place name] issues with your ears isn’t a huge problem in our population … we need lots of funding around diabetes, prevention, management …. It’s not place based funding.” *

 The lack of flexibility in top-down approaches to funding described here goes to the issues of devolved governance, discussed below. Another participant spoke about the duplication of Federal and State governments’ target-funded programs:

*“I think of the waste that’s going on with [Federal and State] departmental staff doing very, very similar jobs, providers being funded to do similar things, only because there’s a political imperative.” *

 An ACCHO that we engaged with as partner organisation in our CTG case study reported having to administer over 90 individual lines of target funding, each with its own reporting demands. Many targeted programs were also seen as ‘deficit’ focused – attempting to remediate problems – rather than being ‘strength-based’ by aiming to build individual or community resources for wellbeing. All of these issues are likely to detract from the value of targeted funding in contributing to equity of access to services.

 Turning to the CTG case study specifically, our analysis of policy structures showed that CTG targets to improve specified health, education and employment outcomes drove implementation of multiple, targeted strategies. These were led by Federal agencies in the health, education and employment portfolios and delivered by public, non-governmental or private for-profit service providers. Consistent with other targeted policies, these strategies were generally decided, funded and regulated in a prescriptive ‘top-down’ manner by government agencies.

 Most CTG case study informants, while not claiming that these various targeted strategies had no value, argued strongly that they were too *limited*, too *unequal* in terms of power relationships, and too *constraining* on the role of Aboriginal and Torres Strait Islander leaders and community-controlled organisations operating locally, to exercise their own choices, capacities and cultural knowledge, to generate community empowerment and wellbeing. As one Aboriginal informant (and leading national policy advocate) described:

*“…while ever we’ve got … ‘Closing the Gap’…it necessarily frames us in the deficit. We should absolutely prosecute the gap but…my expectations are greater than just this overarching vision of eliminating the gap in life expectancy …The vision for me is that we are strong First Nations people and able to prosecute the very best for our kids and our grandkids and their grandkids.”*

 The kinds of additional services and programs that informants saw as needed were community-led, culturally relevant, strength-based strategies to build individual and collective resources for wellbeing. Already-existing examples identified in our research, driven through the work of ACCHOs and other community-led organisations, included (inter alia) youth leadership programs, small business development, cultural knowledge and language programs, caring for country, housing services, and family support.^27^ The value of these strategies in addressing social determinants of Indigenous health was also evident. However, they were often run on the basis of goodwill or limited, insecure funding, and lack consistent structural support and resources.

*“I think one of the ways I’ve seen that sort of issue [of top-down service delivery] dealt with or start to be dealt with is when communities start to set their own agenda. When I was out in [region name] we didn’t ask for anyone’s permission. The communities … they established their own working parties just on the strength of the community’s interests so … we didn’t go to the health department and ask for permission. We didn’t go to any other government department.” *

 Thus, what Aboriginal and Torres Strait Islander people see as required for health and wellbeing certainly incorporates equity of access to affordable, available and culturally safe, acceptable services, but requires these and other strategies to be implemented in ways that systematically strengthen community control, empowerment and self-determination.^27,32,41^ Targeting was still seen by our informants as the appropriate structural approach, but one that requires fundamental change in how it is governed and implemented.^56^

##  Devolved Governance Structures

 Analysis of our findings shows both universal and targeted policies relevant to health equity can be implemented via a top-down construct of policy governance where governments, ministers and central public agencies prescriptively regulate resources and funded services in an attempt to control precisely how public resources are used.^37^ This is what Hill and Hupe describe as a ‘performance management’ approach to policy governance.^37^ However, as noted earlier, literature on multi-level governance^35,36^ recognises practices of devolved governance whereby governments agencies assign some authority to structures operating at a more localised scale to make decisions about how resources are deployed; enabling greater *flexibility* to tailor strategies to suit local conditions and needs.^36^ Evidence on place-based policy suggests such flexibility can be well suited to addressing equitable access to services to meet a diversity of needs at a local scale.^39^

 We identified existing practices of devolved governance in each of our case study areas. In all cases these were bodies operating within a particular geographic region, smaller than a State government jurisdiction. The entities acting as the localised governance body varied between cases. In the PHC and LUP cases they were semi-autonomous organisations established by government. In the CTG case, they were community-controlled organisations created by and within Aboriginal or Torres Strait Islander communities, and in the NBN case it was local governments. In all instances, the flexibility of devolved governance was evident – to exercise authority in order to tailor use of resources to local conditions. In all cases, we found that devolved governance structures could offer benefits for equity of access to social services or built-form/infrastructure resources. However, at the same time continuing structures of top-down, central agency control had the potential to limit or prevent these benefits.

 In our LUP case study, two purpose-built governance structures were created to coordinate infrastructure investments and urban planning activities in the Western Sydney region: a coordinating agency, the Greater Sydney Commission (GSC), and a regional agreement structure, the ‘Western Sydney City Deal’ (WSCD) involving Federal, State and Local government agencies. The investment was relevant to equity of access because it injected new money into a socially and economically disadvantaged urban region in order to address relatively poor access to services and infrastructure such as health and education services, public transport, urban centres providing employment opportunities, and recreational green space.^57^ This investment was seen to respond to years of poor planning:

*“There’s an understanding that there’s been a social, equitable, health and environmental sustainability time bomb created in Western Sydney through a lack of planning.” *

 The governance structures of the GSC and WSCD were authorised and instituted by the State Government, to coordinate a coherent ‘place-making’ approach to investment across these multiple domains. As one informant said, *‘In the GSC itself place-making is a critical element… we’re not just building infrastructure, but we’re building communities that are integrated, linked.’* Thus, the potential equity benefits of a devolved governance approach in this case lay in an ability to lead coordinated action across three levels of government and multiple agencies (which had been lacking) to address multiple issues in a disadvantaged region. However, the project only addressed availability of services and infrastructure not affordability or acceptability, and we saw little evidence of strategies to address inequities in access to planning-related determinants of health *within* the region. There was no involvement of either the public, civil society or non-governmental organisations with a social services remit in the development of the WSCD. This may be because local government was positioned as the lead agency at the local level. However, despite the suggestion that local councils were connected to communities and that the Deal would trickle down to benefit the whole population, the governance lens remained on availability and financing of infrastructure for economic growth rather than affordability or acceptability of infrastructure or service improvements for socially or economically disadvantaged groups within the region. Ratings of LUP policy in Table reflect this assessment.

 In our PHC case study, PHNs were an existing devolved governance structure created for the purpose of assessing needs at a regional scale and tailoring services flexibly to meet those needs. Thus we found that PHNs had significant potential to complement Medicare – the universal PHC system – to improve equity of access to PHC services according to need, within their respective regions. However, at the time of our research, this potential benefit was limited by a top-down, prescriptive approach to targeted funding for PHN activities; restricting the flexibility that is key to devolved governance. As one PHN informant described:

*“Commonwealth [is] giving us funds typically still on a program allocation basis…which again is counterintuitive to a whole-of-population commissioning approach [where…] you would leave us to determine what the community needs were, what the service and system needs were and then look at how we use our funds globally to address those needs.” *

 As noted earlier, PHN funding is allocated according to proxy indicators of need, but the currently limited and prescriptive nature of funding is likely to limit any gains in reducing inequities within or between regions. However, it does indicate the potential of devolved governance structures operating regionally such as PHNs to be a vehicle for proportionality within a universal system; and to counter the inverse proportionality of Medicare-funded primary medical care noted earlier. The fulfilment of these potential benefits of PHNs for equity of access both within and between regions will require funding and regulatory reform, to increase overall funding and enable greater flexibility in how it is applied.^25^ We see potential for gains in the availability and acceptability dimensions that are lacking in the universal Medicare system.

 In our CTG case study, devolved governance was understood in terms of Indigenous governance organisations operating at a local or regional scale having greater authority and flexibility to determine how policy resources are deployed to meet needs within their respective places and communities. ACCHOs and other already-established governance structures (the nature of which varies between communities) were seen as key players. Informants offered many examples of the benefits of such localised control, but also noted how these benefits are restricted by the top-down, fragmented and prescriptive practices of targeted funding.

*“The structural impediments are that funding comes down through appropriation, through budgets into portfolios and then they get heavily protected so the ability to be non-top down or flexible…to respond to community identified priorities…is absolutely desirable but almost impossible to implement.” *

 Devolved governance was also seen as a means by which community-based initiatives could direct resources toward culturally relevant, strength-based strategies, rather than being limited to the predominantly deficit and illness focus of current targeted policies. Finally, devolved governance was also seen as a mechanism for governments to support Aboriginal and Torres Strait Islander peoples’ rights to self-determination. However, governments were still recognised as essential partners in this process. As one Aboriginal informant described:

*“…more and more organisations through their leadership and governing bodies are influencing a really different… relationship with the government representatives on the ground, and seeing government more so as partners now; as community organisations are building their [local] networks…to help respond to some of the community’s priorities.” *

 Thus, devolved governance in this context, vested in Indigenous-led organisations operating at a local or regional scale, has potential to strengthen availability and especially the acceptability of culturally safe services for Aboriginal and Torres Strait Islander communities, tailored to local needs and priorities.

 In Table, we rated CTG policy as positive for equity of access in availability and affordability because of the support for ACCHO services. We rated performance on acceptability as mixed because, although ACCHOs provide culturally safe services, racism remains a barrier to ‘mainstream’ health services and top-down modes of governance and funding are barriers to culturally relevant, strength-based strategies and self-determination at a local scale.

 In our NBN case study, implementation of a universal policy was tightly controlled by Federal agencies and no mechanisms of devolved governance were identified. However, policy actors operating in local government did nevertheless describe how some degree of control of NBN implementation at a local scale could deliver benefit by, again, tailoring the service to meet local needs. One local government actor described how NBN implementation processes (following a prescriptive national plan) had failed to meet community needs in his rural region, and how his organisation had borrowed money and sought additional State government funding to address this problem.^26^

##  Limitations

 We see our theory-informed, inductive and qualitative approach as useful for critically assessing how policy structures work in a particular context and why, therefore, they are likely to have certain effects on equity of access to health-related goods and services. Other forms of research that quantify effects of specific universal or targeted policy interventions on equity of access to such goods or services are likely to complement the kind of research we have presented.

## Discussion

 Debates about the strengths and weaknesses of universal and targeted policies to address equity of access to goods and service relevant to human welfare are longstanding^1^ and are reflected in public health literature.^13,58^ Our research suggests that the ‘right answer’ to optimise equity of access to social services and infrastructure probably does not lie in formulaic commitment to any one model. Instead it suggests that study of policy implementation can be used to identify strengths and weaknesses for issues of availability, affordability and acceptability in these different structural arrangements, in particular contexts. In the Australian case, the evidence suggests that a mix of universal, proportionate and targeted implementation structures, including devolved governance structures, deployed in health and social policy, infrastructure policy and other policy areas, will be best suited to achieve equity in affordability, availability *and* acceptability, and therefore be best-placed to support health equity.

 Our research suggests that the benefits of universal systems for equity of access to services and infrastructure should continue to be understood against the alternative of residualist or purely market-based approaches. Actions by neoliberal governments in Australia to increase the role of PHI in PHC policy^59^ shows that residualist policies continue to be seen by some as a desirable alternative to universalism. However, this research also bears out criticism of universality as a one-size-fits-all approach, which does not necessarily take account of differences in need between population groups. Our NBN and PHC case studies also show that benefits of universality for equity of access may be limited by implementation structures that favour only one dimension of equity and overlook others. Several lessons for equitable universal systems are indicated in our analysis of these two cases. First, the baseline service model adopted in both systems lacked flexibility to address a variety of service user needs and adapt to changes in need over time. Thus, a more robust and flexible baseline would add to the acceptability dimension of universal systems that do reasonably well in affordability and/or availability.^25,26^ In PHC, this would suggest that universal comprehensive PHC would be a more effective baseline model than the current GP-based episodic primary medical care model. In NBN, the originally planned (but not implemented) baseline service using fibre optic cable to reach each premises would have easily met continuing increases in data speed demands.^26^ In our PHC case, the limitations of the baseline service model were addressed to some extent by a complex, somewhat ad hoc array of target-funded services, but a more robust and flexible baseline would reduce demand for these additional expenditures.

 However, systemic inequities in health needs and outcomes between population groups also indicates that, to ensure equity of access, both proportionate-universal and targeted structures must have a place to augment universal systems. Proportionality is conceived as a social policy response to social gradients in health.^22^ Our PHC case suggests that proportional funding based on assessed needs by geographic regions and distributed via PHNs, could be an effective model to apply. At that scale, proportionate funding could be applied flexibly to ‘intensify’ availability, affordability or acceptability of services, as required to meet local needs.

 Targeting, then, is about responding to the needs and goals of particular groups, which are not met adequately by a universal model of service or supply. Targeting can aim to augment the capacities of a universal service to address these needs, or it can be applied to support separate stand-alone services, programs or strategies alongside a universal system. Targeting in either sense is potentially favourable to equity of access, and is quite different to a residualist model of targeting. Our CTG case study suggests that equity of access to services for Aboriginal and Torres Strait Islander people requires both forms of targeting, to improve cultural safety of universal services *and* to support stand-alone community-led services, programs and strategies. ACCHOs provide a CPHC model of service to address a variety of needs in Aboriginal or Torres Strait Islander communities, including needs in terms of both availability and acceptability of services often not met by universal services. Furthermore, targeted funding to stand-alone services and programs has the potential to support Aboriginal or Torres Strait Islander health and wellbeing in ways that go beyond equity of access to a social service as such. (The concept of equity of access ‘according to need’ has potential limitations in this sense, if need is operationalised purely in terms of provision of interventions to ‘fix’ specified problems such as an ill-health condition). As discussed earlier, this is about targeted funding support as a vehicle for culturally relevant strength-based strategies, community empowerment and self-determination^27,32,56^; processes that address social determinants of Indigenous health such as having a sense of control^60^ and a strong connection to culture.^61^ In a sense, the CTG case study exposed a gap between government agencies’ approach to targeting as ‘delivery of top-down interventions to fix problems’ and our Aboriginal and Torres Strait Islander informants’ aims to shift targeted policy onto a different conceptual and operational basis, to support self-determination.

 Triangulation of results across our PHC, CTG and NBN case studies indicated that devolved governance structures have a useful role to play in achieving equity of access to goods and services relevant to health. It appears that, in Australia, policy-makers are still ‘experimenting’ with devolved governance in the sectors studied; possibly preferring purpose-specific organisational structures sitting alongside the ‘mainstream,’ three-tiered Federal structure. Other national jurisdictions may have a two-tiered structure, or local governments may have a stronger role in governing policy delivery. In any case, our findings bear out the key theorised value of devolved governance structures, to be able to implement policies in ways that respond *flexibly* to various community needs within a particular locale. Our PHC and NBN case suggest that use of devolved governance structures operating regionally (eg, PHNs or local governments) could be an effective means to implement universal policy; supporting equity of access by responding to local needs in relation to availability, affordability or acceptability.

 Similarly, our CTG case study indicates that devolved governance at a regional or local scale can play a role in effective implementation of targeted policies, again by flexibly tailoring actions to meet local communities’ needs and goals. A systemic shift to use of such structures could overcome some of the aforementioned weaknesses of targeted funding practices such as short-termism, duplication and excessive regulatory demands. Aboriginal and Torres Strait Islander leaders we spoke to want such structures to be authorised and funded to go beyond oversight of ‘conventional’ service delivery and advance strength-based, community-engaged strategies for localised social, economic and cultural development. These aims speak to the broad vision of health promotion articulated in the Ottawa Charter, and in this sense our findings on devolved governance may hold lessons for implementation of health promotion strategies more broadly,^24^ and support existing programs like Healthy Cities (implemented in many countries across the globe) and Health in All Polices implemented at a state or provincial level in US, Canada and Australia.

## Conclusion

 Within on-going debates about the merits of universal, proportionate-universal or targeted policies as structural mechanisms to improve health equity, we conclude that all potentially have a role to play, depending on the context in which they’re applied. Rather that choosing one or other strategy, policy-makers would be well advised to seek a combination of approaches. Devolved governance structures operating regionally have potential to strengthen the equity benefits of both universal and targeted approaches, but will require significant reform of top-down, prescriptive approaches to policy governance.

## Acknowledgements

 We would like to acknowledge and thank our colleagues on the Centre of Research Excellence on the Social Determinants of Health Equity research team, members of the project’s Critical Policy Reference Group, and all those who participated in the research.

## Ethical issues

 The research reported was approved by the Flinders University Social and Behavioural Research Ethics Committee.

## Competing interests

 MF reports grants from National Health and Medical Research Council, during the conduct of the study. Other authors declare that they have no competing interests.

## Authors’ contributions

 MF led case study research, contributed to synthesis of findings and led writing of the paper. PH led case study research, contributed to synthesis of findings and reviewed draft versions of the paper. TF contributed to case study research, contributed to synthesis of findings and reviewed draft versions of the paper. TM led case study research, contributed to synthesis of findings and reviewed draft versions of the paper. EG contributed to case study research, contributed to synthesis of findings and reviewed draft versions of the paper. SF co-led the research team, contributed to synthesis of findings and reviewed draft versions of the paper. FB co-led the research team, contributed to synthesis of findings and reviewed draft versions of the paper.

## Funding

 The research reported in this article was conducted through the Centre of Research Excellence on the Social Determinants of Health Equity, funded by the Australian National Health and Medical Research Council (GNT1078046). The funder had no role in: study design; collection, analysis and interpretation of data; writing of the article; or the decision to submit it for publication.

## References

[1] Ellison N (1999). Beyond universalism and particularism: rethinking contemporary welfare theory. Crit Soc Policy.

[2] Bergh A (2004). The universal welfare state: theory and the case of Sweden. Polit Stud.

[3] Mkandawire T. Targeting and Universalism in Poverty Reduction. Geneva: United Nations Research Institute for Social Development; 2005.

[4] Sanders D, Nandi S, Labonté R, Vance C, Van Damme W (2019). From primary health care to universal health coverage-one step forward and two steps back. Lancet.

[5] Carey G, Crammond B, De Leeuw E (2015). Towards health equity: a framework for the application of proportionate universalism. Int J Equity Health.

[6] Commission on Social Determinants of Health. Closing the Gap in a Generation: Health Equity Through Action on the Social Determinants of Health. Geneva: World Health Organization; 2008. 10.1016/S0140-6736(08)61690-6 18994664

[7] Baum F, Friel S (2017). Politics, policies and processes: a multidisciplinary and multimethods research programme on policies on the social determinants of health inequity in Australia. BMJ Open.

[8] Public Health Information Development Unit. Monitoring Inequality in Australia. http://phidu.torrens.edu.au/social-health-atlases/graphs/monitoring-inequality-in-australia/whole-population. Accessed December 15, 2020. Published 2020.

[9] Chetty R, Stepner M, Abraham S (2016). The association between income and life expectancy in the United States, 2001-2014. JAMA.

[10] Bremberg S (2020). Rural-urban mortality inequalities in four Nordic welfare states. Scand J Public Health.

[11] Woolf SH, Schoomaker H, Hill L, Orndahl CM (2019). The social determinants of health and the decline in US life expectancy: implications for Appalachia. J Appalach Health.

[12] Beveridge W. Social Insurance and Allied Services. London: The Stationery Office; 1942.

[13] Carey G, Crammond B (2017). A glossary of policy frameworks: the many forms of ‘universalism’ and policy ‘targeting. ’ J Epidemiol Community Health.

[14] Harvey D. A Brief History of Neoliberalism. Oxford: Oxford University Press; 2005.

[15] Raphael D (2013). The political economy of health promotion: part 1, national commitments to provision of the prerequisites of health. Health Promot Int.

[16] Baum F. Governing for Health: Advancing Health and Equity Through Policy and Advocacy. Oxford: Oxford University Press; 2019.

[17] Rose G (1985). Sick individuals and sick populations. Int J Epidemiol.

[18] Lorenc T, Petticrew M, Welch V, Tugwell P (2013). What types of interventions generate inequalities? evidence from systematic reviews. J Epidemiol Community Health.

[19] Williams F. Somewhere over the rainbow: universality and diversity in social policy. In: Manning N, Page R, eds. Social Policy Review. Canterbury: Social Policy Association; 1992. p. 200-219.

[20] Reynolds AJ, Temple JA, White BA, Ou SR, Robertson DL (2011). Age 26 cost-benefit analysis of the child-parent center early education program. Child Dev.

[21] Davy C, Harfield S, McArthur A, Munn Z, Brown A (2016). Access to primary health care services for Indigenous peoples: a framework synthesis. Int J Equity Health.

[22] Marmot M, Allen J, Goldblatt P, et al. Fair Society, Health Lives: Strategic Review of Health Inequalities in England Post-2010. London: U.K. Department of Health; 2010.

[23] NHS Scotland. Proportionate Universalism and Health Inequalities. Edinburgh, Scotland: NHS Scotland; 2014.

[24] de Leeuw E (2017). Engagement of sectors other than health in integrated health governance, policy, and action. Annu Rev Public Health.

[25] Fisher M, Freeeman T, Mackean T, Friel S, Baum F. Universal Health Coverage for non-communicable diseases and health equity: Lessons from Australian Primary Health Care. Int J Health Policy Manag 2020; 10.34172/ijhpm.2020.232. PMC9309940 33300769

[26] Fisher M, Freeman T, Schram A, Baum F, Friel S (2020). Implementing policy on next-generation broadband networks and implications for equity of access to high speed broadband: A case study of Australia’s NBN. Telecomms Policy.

[27] Fisher M, Mackean T, George E, Friel S, Baum F (2021). Stakeholder perceptions of policy implementation for Indigenous health and cultural safety: A study of Australia’s ‘Closing the Gap’ policies. Aust J Public Admin.

[28] Harris P, Kent J, Sainsbury P, Riley E, Sharma N, Harris E (2019). Healthy urban planning: an institutional policy analysis of strategic planning in Sydney, Australia. Health Promot Int.

[29] Gardiner FW, Bishop L, de Graaff B, Campbell JA, Gale L, Quinlan F. Equitable Patient Access to Primary Healthcare in Australia. Canberra: Royal Flying Doctor Service; 2020.

[30] Anderson I, Baum F, Bentley M. Beyond Bandaids: Exploring the Underlying Social Determinants of Aboriginal Health. Darwin: Cooperative Research Centre for Aboriginal Health; 2007.

[31] Yin RK. Case Study Research: Design and Methods. 4th ed. London: SAGE Publications; 2009.

[32] Mackean T, Fisher M, George E, Friel S, Baum F. Improving implementation of Closing the Gap policy to support Aboriginal and Torres Strait Islander sovereignty and wellbeing. Lowitja Institute Indigenous Health and Wellbeing Conference; June 18-20, 2019; Darwin.

[33] Solar O, Irwin A. A Conceptual Framework for Action on the Social Determinants of Health. Geneva: World Health Organization; 2010.

[34] Howlett M, Ramesh M, Perl A. Studying Public Policy: Policy Cycles & Policy Subsystems. Toronto: Oxford University Press; 2009.

[35] Alcantara C, Morden M (2019). Indigenous multilevel governance and power relations. Territ Politic Gov.

[36] Hooghe L, Marks G (2003). Unraveling the central state, but how? types of multi-level governance. Am Polit Sci Rev.

[37] Hill M, Hupe P. Implementing Public Policy: An Introduction to the Study of Operational Governance. 3rd ed. London: SAGE Publications; 2014.

[38] Bradford N. Place-Based Public Policy: Towards a New Urban and Community Agenda for Canada. Ottawa: Canadian Policy Research Networks; 2005.

[39] Dankwa-Mullan I, Pérez-Stable EJ (2016). Addressing health disparities is a place-based issue. Am J Public Health.

[40] Goldstein G (2000). Healthy cities: overview of a WHO international program. Rev Environ Health.

[41] Mackean T, Fisher M, Friel S, Baum F (2019). A framework to assess cultural safety in Australian public policy. Health Promot Int.

[42] Crotty M. The Foundations of Social Research: Meaning and Perspective in the Research Process. Sydney: Allen & Unwin; 1998.

[43] Harris MF, Harris E, Roland M (2004). Access to primary health care: three challenges to equity. Aust J Prim Health.

[44] Braveman P, Gruskin S (2003). Defining equity in health. J Epidemiol Community Health.

[45] Levesque JF, Harris MF, Russell G (2013). Patient-centred access to health care: conceptualising access at the interface of health systems and populations. Int J Equity Health.

[46] Thiede M, Akweongo P, McIntyre D. Exploring the dimensions of access. In: McIntyre D, Mooney G, eds. The Economics of Health Equity. Cambridge: Cambridge University Press; 2007. p. 103-123.

[47] Leseman PPM, Slot PL (2020). Universal versus targeted approaches to prevent early education gaps The Netherlands as case in point. Z Erziehwiss.

[48] Menadue J. Propping up Private Health Insurance is Like Putting Lipstick on a Pig. Surry Hills, NSW: The Guardian Australia; 2017.

[49] Schram A, Friel S, Freeman T, Fisher M, Baum F, Harris P (2018). Digital infrastructure as a determinant of health equity: an australian case study of the implementation of the National Broadband Network. Aust J Public Adm.

[50] Hajizadeh M, Connelly LB, Butler JR (2012). Health policy and horizontal inequities of health-care utilization in Australia: 1983-2005. Appl Econ Lett.

[51] Korda RJ, Banks E, Clements MS, Young AF (2009). Is inequity undermining Australia’s ‘universal’ health care system? socio-economic inequalities in the use of specialist medical and non-medical ambulatory health care. Aust N Z J Public Health.

[52] Alsharif AT, Kruger E, Tennant M (2014). Disparities in dental insurance coverage among hospitalised Western Australian children. Int Dent J.

[53] Chrisopoulos S, Luzzi L, Brennan DS (2013). Trends in dental visiting avoidance due to cost in Australia, 1994 to 2010: an age-period-cohort analysis. BMC Health Serv Res.

[54] Duckett S, Swerissen H, Moran G. Building Better Foundations for Primary Care. Melbourne: Grattan Institute; 2017.

[55] Australian Institute of Health and Welfare (AIHW). Australian Health Expenditure by Remoteness: A Comparison of Remote, Regional and City Health Expenditure. Canberra: AIHW; 2011.

[56] Empowered Communities. Empowered Communities: Empowered Peoples Design Report. Canberra: Wunan Foundation Inc; 2015.

[57] Australian Government, NSW Government, Blue Mountains City Council, et al. Smart Cities Plan - Implementation: Western Sydney City Deal. Sydney; 2018. https://www.infrastructure.gov.au/sites/default/files/migrated/cities/city-deals/western-sydney/files/western-sydney-city-deal-implementation-plan.pdf.

[58] Brennenstuhl S, Quesnel-Vallée A, McDonough P (2012). Welfare regimes, population health and health inequalities: a research synthesis. J Epidemiol Community Health.

[59] Windle A, Fisher M, Freeman T (2018). Increased private health fund involvement in Australia’s primary health care: Implications for health equity. Aust J Soc Issues.

[60] Tsey K (2008). The control factor: a neglected social determinant of health. Lancet.

[61] Dockery AM (2010). Culture and wellbeing: the case of Indigenous Australians. Soc Indic Res.

